# Derivation of iPSCs after Culture of Human Dental Pulp Cells under Defined Conditions

**DOI:** 10.1371/journal.pone.0115392

**Published:** 2014-12-18

**Authors:** Tomoko Takeda-Kawaguchi, Ken Sugiyama, Shunji Chikusa, Kazuki Iida, Hitomi Aoki, Naritaka Tamaoki, Daijiro Hatakeyama, Takahiro Kunisada, Toshiyuki Shibata, Noemi Fusaki, Ken-ichi Tezuka

**Affiliations:** 1 Department of Oral and Maxillofacial Science, Gifu University Graduate School of Medicine, Gifu, Japan; 2 Department of Tissue and Organ Development, Gifu University Graduate School of Medicine, Gifu, Japan; 3 Department of Ophthalmology, Keio University School of Medicine, Tokyo, Japan; Instituto Butantan, Brazil

## Abstract

Human dental pulp cells (hDPCs) are a promising resource for regenerative medicine and tissue engineering and can be used for derivation of induced pluripotent stem cells (iPSCs). However, current protocols use reagents of animal origin (mainly fetal bovine serum, FBS) that carry the potential risk of infectious diseases and unwanted immunogenicity. Here, we report a chemically defined protocol to isolate and maintain the growth and differentiation potential of hDPCs. hDPCs cultured under these conditions showed significantly less primary colony formation than those with FBS. Cell culture under stringently defined conditions revealed a donor-dependent growth capacity; however, once established, the differentiation capabilities of the hDPCs were comparable to those observed with FBS. DNA array analyses indicated that the culture conditions robustly altered hDPC gene expression patterns but, more importantly, had little effect on neither pluripotent gene expression nor the efficiency of iPSC induction. The chemically defined culture conditions described herein are not perfect serum replacements, but can be used for the safe establishment of iPSCs and will find utility in applications for cell-based regenerative medicine.

## Introduction

Human dental pulp cells (hDPCs) can be obtained from human third molar teeth and contain abundant dental pulp stem/progenitor cells [1]–[3]. Previously, we isolated hDPCs from more than 180 patients, most of who were young and had wisdom teeth undergoing maturation; these cells are easily handled, highly proliferative, and can be stored in liquid nitrogen for extended periods using conventional procedures [3] [4]. However, hDPC culture protocols involve the use of fetal bovine serum (FBS), which is associated with a variety of quality control and safety issues. The composition of animal serum is unknown and varies between batches, interfering with the reproducibility of experiments; moreover, sera be contaminated with viruses, mycoplasma, prions or other pathogenic, toxic or immunogenic agents [5]–[11]. Although little is known regarding other xenogeneic products, such as porcine-derived trypsin that is used for detaching cultured cells, they are likely to carry similar biosafety risks. To avoid these risks, several culture methods have been proposed to establish somatic cells for clinical use; however, these protocols use human serum in place of FBS [12] [13]. The objective of our research was to design and evaluate a protocol to isolate, expand, and maintain clinically safe and efficient hDPCs by completely removing serum and replacing it with a novel mixture composed primarily of chemically defined materials.

One of our goals is to establish a set of hDPC lines homozygous for human leukocyte antigen (HLA) haplotypes. We previously reported that retroviral transduction of four transcription factors (*OCT3/4*, *SOX2*, *KLF4*, and *c-MYC*) can reprogram DPCs into induced pluripotent stem cells (iPSCs) that closely resemble embryonic stem cells (ESCs). These findings suggested that a significant number of iPS cell lines homozygous for HLA haplotypes can be established from hDPCs, and are thus a valuable resource for regenerative medicine [14] [15]. HLA matched iPSCs are a potential source of patient-specific pluripotent stem cells that could be used to treat a number of human degenerative diseases without evoking immune rejection. The risk of oncogene activation and other genomic perturbations caused by retroviral integration during iPSC generation also needs to be addressed before ESCs and iPSCs can be safely used for clinical cell therapy. As it is an episomal vector, Sendai virus can be used avoid the risk of integration of the c-MYC oncogene into host genome [16] [17] during reprogramming. With this in mind, we incorporated Sendai virus into our novel method for derivation of iPSCs from hDPCs grown in defined chemical conditions.

## Materials and Methods

### Ethics Statement

Human dental pulp tissue collection, subsequent iPS cell generation and genome/gene analyses performed in this study was approved by the Ethics Committee of Gifu University (approval number: 22–280). Written informed consent was obtained from each individual patient and guardians in case the patient was under 20 years. All animal experiments in this study strictly followed a protocol approved by the Institutional Animal Care and Use Committee of Gifu University (approval number: 24-18).

### Isolation and Culture of Human Dental Pulp Cells

Using a protocol approved by the Institutional Review Board of Gifu University, we collected normal human third molars at the Gifu University Medical Hospital after having obtained informed consent from each patient (age, 14 years). The pulp tissues were minced into small clumps and digested with 1 mg/ml collagenase type I (Sigma-Aldrich, Saint Louis, MO, USA) or ACCUTASE (Funakoshi, Tokyo, Japan) for 30 min at 37°C. Several colonies were obtained after these small clumps were transferred to culture dishes containing MSCGM medium (Luna, Walkersville, MD, USA) or SERICIN GIT medium (Wako, Osaka, Japan), MF-medium (Toyobo, Osaka, Japan), STK2 medium (DS Pharma, Osaka, Japan), and MSCGM-CD medium (Lonza). Fibroblastic cells that subsequently grew out from these colonies were expanded at 37°C in a humidified atmosphere of air (21% O_2_) and 5% CO_2_ in MSCGM or MSCGM-CD medium. For *in vitro* differentiation experiments, we used at least 3 independent cell lines for each group. For the induction of osteo/odontogenesis, hDPCs were cultured in MSCGM medium supplemented with 0.1 µM dexamethasone, 50 µg/ml ascorbic acid (Wako), and 0.1% β-glycerophosphate (Sigma-Aldrich). hDPCs were plated at 1.0×10^5^ cells/well in 12-well plates. After a culture period of 15 d, alkaline phosphatase (ALP) analysis was performed with an ALP staining kit (Muto, Tokyo, Japan). For the induction of adipogenesis, an adipogenic induction medium kit (Lonza) was used according to the manufacturer's instructions. We assessed the accumulation of neutral lipid-containing vacuoles by staining with Oil Red O.

To determine the number of cumulative population doublings, we seeded hDPCs into 10-cm dishes. Every 3–5 d, when the cells had become confluent, they were digested with 0.05% trypsin-EDTA (Life Technologies, Carlsbad, CA, USA) or ACCUTASE and re-seeded at a density of 1.0×10^6^ cells/dish. This procedure was repeated for every passage (P). Cells were counted at each passage with a hemocytometer.

### Transplantation and Preparation of Histological Sections

Approximately 1.0×10^6^ hDPCs were seeded onto an Atelocollagen coated β-TCP scaffold (KOKEN, Tokyo, Japan), cultured for 7 d, and then transplanted subcutaneously into the dorsal surfaces of 8-week-old immunodeficient nude mice (BALB/c nu/nu; CLEA, Tokyo, Japan), as previously described [3] [4]. Our animal use protocols were reviewed and approved by the Gifu University Institutional Review Board. The transplants were removed at 12 weeks post-transplantation, and fixed with phosphate buffered saline (PBS) containing 4% paraformaldehyde. The transplants were embedded in resin (OsteoResin Embedding kit, WAKO). Resin-embedded tissues were sectioned (6 µm in thickness) using a tungsten carbide bur (SH35W, FEATHER, Osaka, Japan) and stained with hematoxylin and eosin. Digital images were analyzed using ImageJ software (National Institutes of Health). For the digital image analysis, seven holes from the same composite were randomly selected for analysis. The average percentage of dentin area relative to the holes area of each implant was calculated.

### cDNA Microarray Analysis

hDPCs from three donors (DP245, DP264, and DP265) were cultured in MSCGM or MSCGM-CD medium. Total RNA was isolated from the cells with an RNeasy Plus Mini Kit (Qiagen, Valencia, CA, USA). After RNA was quantified using an Agilent 2100 Bioanalyzer, 250 ng of total RNA was converted to cDNA, amplified, and labeled with Cy3-labeled CTP using the Low Input Quick Amp Labeling kit (Agilent Technologies, Santa Clara, CA) according to the manufacturer's protocol. Following labeling and clean-up, the cDNAs were quantified using an ND-1000 Spectrophotometer (Nano Drop Technologies, Wilmington, DE) and hybridized with a whole human genome 4×44 K oligo-DNA microarray (Agilent Technologies). After hybridization, the arrays were washed consecutively using Gene Expression Wash Pack (Agilent Technologies). Fluorescence images of the hybridized arrays were generated using the Agilent DNA Microarray Scanner, and the intensities were determined using Agilent Feature Extraction software ver.10.7.3.1. Each sample was analyzed once. The level of gene expression was determined using Gene Spring GX11.5 (Agilent Technologies). Microarray data have been deposited in the NIH Gene Expression Omnibus database under the accession number GSE59498.

### Cell Culture and Generation of iPSCs

We generated iPSCs from hDPCs cultured in MSCGM or MSCGM-CD medium according to a published procedure [14] [16] [17]. Following the guidelines for the generation of human iPSCs approved by the Institutional Review Board of Gifu University, we used hDPCs from two donors (DP264 and DP265, 14-year-old girl) for generating iPSCs within 10 passages. Using a Sendai viral vector kit (DNAVEC, Tsukuba, Japan), we generated iPSCs that express the transcription factors encoded by *OCT3/4*, *SOX2*, *KLF4*, and *c-MYC* according to a published protocol [16] [17]. We first introduced the Sendai viruses into hDPCs according to the manufacturer's instructions. hDPCs were seeded at 5.0×10^5^ cells/6 well and cultured in MSCGM or MSCGM-CD medium. Beginning the day after Sendai viral infection, the culture media were changed daily up to day 7. On day 7 post-transduction, the cells were trypsinized using 0.05% trypsin-EDTA (Life Technologies), and hDPCs (2.0×10^4^, 3.0×10^4^, 5.0×10^4^ cells/6 cm dish) were seeded onto mitomycin C-treated SNL feeder layers (SNL cell line obtained from Sanger Institute, Cambridge, UK) in Primate ES cell medium (ReproCell, Tokyo, Japan) supplemented with 4 ng/ml basic fibroblast growth factor (Wako), and cultured for a further 14 days. On day 21 post-transduction, we counted the number of human ESC-like and alkaline phosphatase (ALP) -positive colonies, and isolated total RNA from the cells to determine the expression level of various genes. For characterization of iPSCs, ESC-like colonies were picked from days 21 to 24 and cultured. A human ESC line (KhES01) was obtained from Kyoto University (Kyoto, Japan) and cultured on mitomycin C-treated SNL feeder layers in Primate ES cell medium (ReproCell) supplemented with 4 ng/ml basic fibroblast growth factor (Wako).

### ALP staining

Cells were fixed with 4% paraformaldehyde for 15 min at room temperature, washed with PBS, followed by 0.1 M Tris-HCl (pH 8.0) containing 50 mM MgCl_2_, and then treated with 0.3 M Tris-HCl (pH 8.0) containing 50 mM MgCl_2_, 0.06% Naphthol AS-MX Phosphate (Sigma-Aldrich) and 0.01% Fast Red Violet (Sigma-Aldrich) for 30 min at room temperature. Reactions were protected from light.

### Immunohistochemistry

The cells were fixed with 4% paraformaldehyde for 15 min and treated with PBS containing 2% normal goat serum (Wako), 0.5% BSA (Sigma-Aldrich), and 0.2% Triton X-100 (Wako) for 30 min. Antibodies specific for the following markers were used in this study: SSEA1 (1∶50), SSEA4 (1∶50), TRA1-60 (1∶50), and TRA1-81 (1∶50, ES characterization kit, Merck Millipore, Billerica, MA, USA). The following secondary antibodies were used: Alexa Fluor 488 labeled anti-mouse IgG (1: 200, Merck Millipore), and Alexa Fluor 488 labeled anti-mouse IgM (1∶200, Merck Millipore). For immunostaining for ESC-specific cell markers, we used ESCs characterized previously [14] as positive controls and incubated samples without primary antibodies as negative controls. Nuclei were stained with 4′, 6-diamidino-2-phenylindole (DAPI) (Merck Millipore).

### Quantitative Real-time Polymerase Chain-reaction (RT-PCR)

RNA extraction (RNeasy Plus Mini Kit; Qiagen) and RT-PCR (Thermal Cycler Dice Real Time System TP800; Takara, Shiga, Japan) were performed as described previously [3] [4] [18]. The mRNA data were normalized to those of *GAPDH* and used to calculate expression coefficients. Primer sequences are shown in S1 Table. Primers used for *OCT3/4*, *SOX2*, and *KLF4* specifically detect the endogenous transcripts [19].

### Teratoma Formation Assays

Teratoma formation assays were performed as reported [19] [20]. Briefly, 3×10^5^ iPSCs were injected using a Hamilton syringe into the testes of six-week-old immunodeficient nude mice (BALB/c nu/nu; CLEA). Twelve weeks after injection, tumors were dissected and fixed with PBS containing 4% paraformaldehyde. Paraffin-embedded tissues were sectioned (3 µm) using a microtome (TU-213, YAMATO KOUKI, Asaka, Japan) and stained with hematoxylin and eosin.

### Statistical Analysis

Data are presented as the mean ± SD. The differences in mean values were evaluated using the *t*-test after evaluation of variances (Microsoft Excel). For RT-PCR, the mean and standard deviation of the expression coefficient were calculated using the Thermal Cycler Dice Real Time System Software Ver. 4.02 (Takara).

## Results

### Isolation and Characterization of hDPCs under Chemically Defined Culture

First, we examined the growth of hDPC cell lines, which we established before [3] using a chemically defined culture method (MF-medium, SERICIN GIT, STK2 and MSCGM-CD condition). The growth of hDPCs in MF-medium and SERICIN GIT was significantly less than those grown in the presence of FBS (data not shown). hDPCs grown in STK2 medium formed significantly less colonies than those grown in the presence of FBS (S1 Figure). Furthermore, when the hDPCs were established in culture, they also grew less rapidly than those grown in FBS-containing medium (data not shown). In contrast, the growth of hDPCs established in MSCGM-CD was comparable to growth in the presence of FBS. Therefore, MSCGM-CD medium was selected for all subsequent experiments.

**Figure 1:**
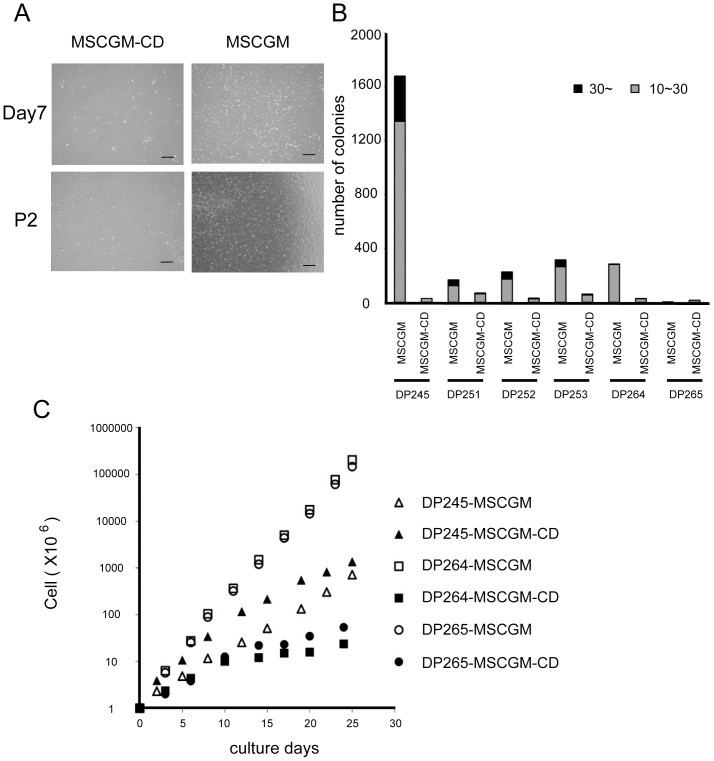
Morphology and growth capability of hDPCs cultured in MSCGM or MSCGM-CD medium. (A) Images of cultured hDPCs (DP245) 7 days after seeding and passage (P) 2. Cells were obtained from the same donor and cultured with MSCGM or MSCGM-CD, respectively (n = 6 donors). Scale bar = 400 µm. (B) Number of colonies derived from cells cultured in MSCGM or MSCGM-CD. hDPCs grown in MSCGM showed significantly higher primary colony formation than those grown in MSCGM-CD. (C) Curves for long-term growth obtained from 6 cell lines cultured with MSCGM or MSCGM-CD medium. hDPCs cultured in MSCGM medium maintained a high growth rate, whereas those in MSCGM-CD had a lower growth rate with the exception of the DP245 cell line.

Dental pulp tissues were isolated from the third molars of 6 females, all of whom were 14 years old (DP245, DP251, DP252, DP253, DP264 and DP265). Tissues were placed into MSCGM-CD medium and hDPCs were successfully isolated from all samples. On culture dishes, these cells formed colonies with a scattered morphology after 7 days' incubation with MSCGM (Fig. 1A). On the other hand, the shape of colonies grown in MSCGM-CD was compact and rounder than MSCGM condition. Thereafter, the morphology of the cells became more spindle-like, and was indistinguishable from those with MSCGM after passage 2 (P2) (Fig. 1A).

hDPCs grown in the presence of FBS (the ‘MSCGM’ medium), showed significantly higher primary colony formation than those grown in MSCGM-CD medium (Fig. 1B). In addition to this initial assay, we then compared the long-term growth curves of the MSCGM and MSCGM-CD groups (Fig. 1C). DP264 and DP265 cultured in MSCGM media maintained a higher growth rate when compared to their growth in MSCGM-CD, whereas DP245 showed a comparable or slightly higher growth rate under in MSCGM-CD medium. However, the growth rate of all lines in MSCGM-CD gradually decreased with the number of passages.

### Differentiation Potential of hDPC Lines Established under Chemically Defined Conditions

**Figure 2:**
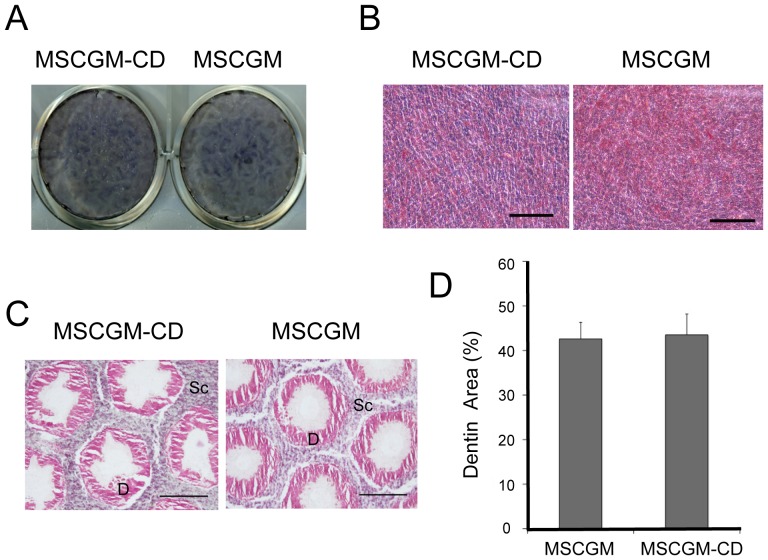
Differentiation ability of hDPCs cultured in MSCGM or MSCGM-CD media. (A, B) *In vitro* differentiation of hDPCs. hDPCs were cultured in MSCGM or MSCGM-CD and then tested for their ability to differentiate into osteo/odontoblastic and adipocyte lineages. (A) Osteo/odontogenesis was assessed by staining for ALP activity. (B) Adipogenesis was assessed by Oil Red O staining. Scale bar = 100 µm. (C, D) *In vivo* differentiation of hDPCs at P5. Transplants were removed at 12 weeks post-transplantation, sectioned, and stained with hematoxylin and eosin (C). In P5 transplants, the scaffolds (SC) were lined with a layer of dentin-like matrix (D). Scale bar = 200 µm. (D) Image analysis of the regenerated dentin area in subcutaneous composites at 12 weeks post-transplantation. The ratio of dentin regenerated in the hole (n = 7) was determined by histomorphometric analysis of the sections and calculated with ImageJ software.

Real-time PCR analysis showed that the expression of osteoblastic marker genes, such as *BGLAP*, *RUNX2*, and *SPP1*, was moderately upregulated in hDPCs cultured in MSCGM-CD medium (S3 Figure). We then cultured cells isolated under serum and chemically defined conditions in medium that promotes osteo/odontoblastic differentiation. After 15 d in culture, the cells became ALP-positive (Fig. 2A). When cells grown in either MSCGM or MSCGM-CD were subjected to a pro-adipocyte milieu, lipid-containing vesicles were observed within 4 weeks (Fig. 2B). At 8 weeks post-transplantation in nude mice, cells established in both MSCGM-CD and MSCGM media started to generate a thick matrix on the surface of the implanted scaffolds, and by 12 weeks post-transplantation, they had formed dentin-like tissues (Fig. 2C). There was no significant difference in the area of calcified matrix between cells derived from MSCGM and MSCGM−CD media (Fig. 2D).

These results suggest that hDPCs isolated in MSCGM-CD medium have a comparable differentiation potential to those established in the classical MSCGM medium.

We also performed chromosome G-banding analysis; this revealed that hDPCs cultured in MSCGM-CD conditions have normal chromosome number and structure (S2 Figure).

### Induction of iPSCs from hDPCs grown in Chemically Defined Media

**Figure 3:**
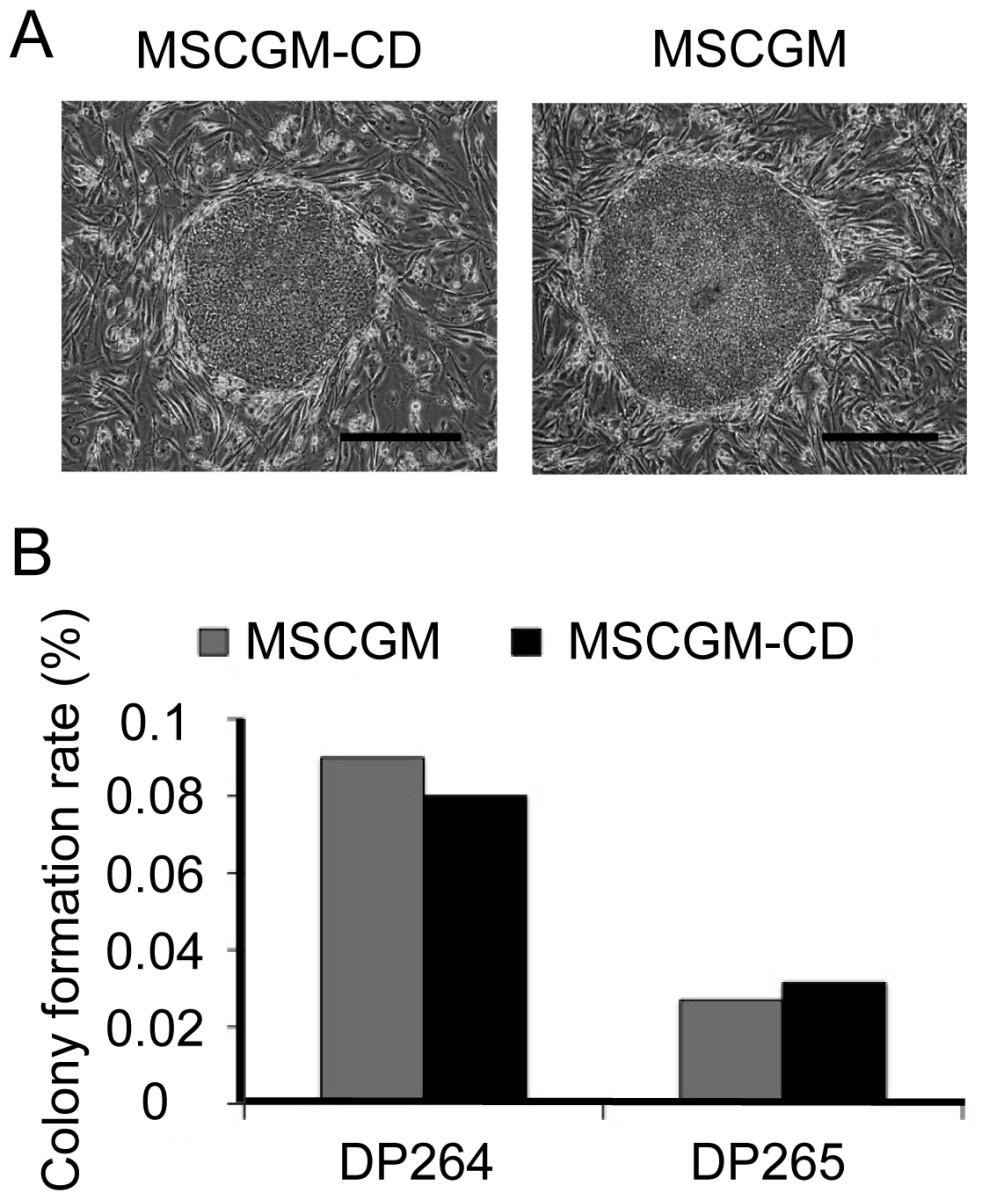
Efficient generation of iPSCs derived from cells cultured in MSCGM or MSCGM-CD media. (A) Typical morphology of an iPSC colony generated from DP264 grown in MSCGM (DP264-M-iPS-5) and MSCGM-CD (DP264-CD-iPS-2). Scale bar = 200 µm. (B) Numbers of ESC-like colonies generated from 3×10^4^ hDPCs on day 21 after Sendai viral transduction. We transduced hDPCs from two donors (DP264, and DP265) with Sendai viral expression vectors containing the genes encoding four reprogramming factors (*OCT3/4, SOX2, KLF4,* and *c-MYC*), and cultured them in MSCGM or MSCGM-CD for 7 days after virus infection. Cells were then reseeded onto SNL feeder cells and incubated for 21 days. Mean numbers of colonies from two experiments (n = 2) are shown.

Using a Sendai virus vector system, *OCT3/4*, *SOX2*, *KLF4*, and *c-MYC* were introduced into hDPCs isolated from two individuals (DP264 and DP265). Several ES-like colonies began to appear in hDPCs by 21 d post infection (Fig. 3A). The number of iPSC colonies was not significantly different between cells grown in MSCGM and MSCGM-CD media (Fig. 3B).

**Figure 4:**
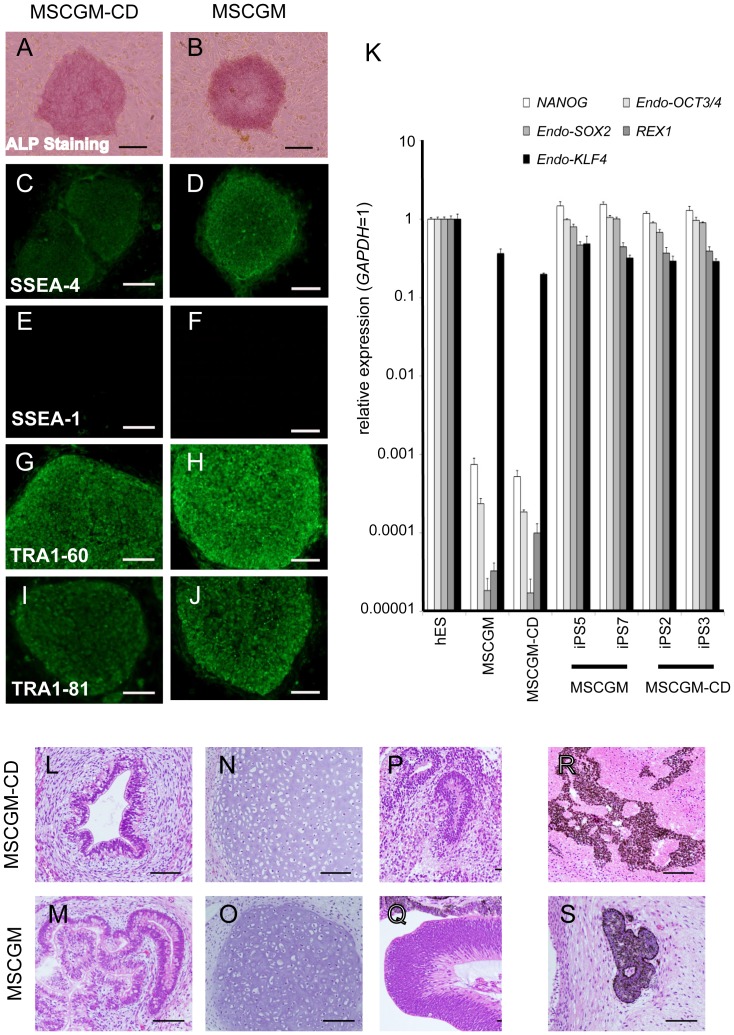
Characterization of iPSCs derived from cells grown in MSCGM-CD or MSCGM. (A–J) iPSCs generated from DP264 grown in MSCGM-CD (D264-CD-iPS-2) and MSCGM (D264-M-iPS-5) expressed ALP (A, B) and pluripotency markers SSEA-4 (C, D), TRA1-60 (G, H), and TRA1-81 (I, J), but not SSEA-1 (E, F), as judged by immunostaining. Scale bar = 200 µm. (K) RT-PCR analysis of ESC-marker genes in iPSCs (D264-CD-iPS) generated from DP264 grown in MSCGM-CD, iPSCs (D264-M-iPS) derived from cells in MSCGM, human ESCs, and DP264. Numbers indicate different iPSC clones generated from DP264. Endogenous *NANOG*, *OCT3/4*, *SOX2*, *REX1*, and *KLF4* were expressed in two iPSC lines derived from cells grown in MSCGM-CD medium, as well as in human ESCs and iPSCs derived from cells grown in MSCGM, but not in DPCs. Error bars indicate the SD calculated from triplicates. (L–S) To confirm the pluripotency of iPSCs generated from DP264 grown in MSCGM-CD, we injected the cells into the testes of immunodeficient nude mice. Twelve weeks after injection, we observed tumor formation. Hematoxylin and eosin-stained teratoma sections show that the tumor contained various types of tissues, such as gut-like epithelial tissues (L, M, endoderm tissue), cartilage (N, O, mesoderm tissue), neural-tube-like structures (P, Q, ectoderm tissue), and pigment cells (R, S, ectoderm tissue). Scale bar = 100 µm.

Each iPSC clone was characterized for ESC-like morphology and ALP activity (Figs. 4A and 4B and S2 Table). They were also positive for SSEA-4, TRA1-60, and TRA1-81, and negative for SSEA-1 (Figs. 4C-4J and S2 Table). RT-PCR analysis showed that expression of *NANOG* and *REX1* was comparable to that of human ESCs, as well as *OCT3/4*, *SOX2*, and *KLF4* (Fig. 4K). These cells also formed teratomas in nude mice, suggesting differentiation towards all three germ layers (Figs. 4L-4S). Karyotypes of iPSC clones established in MSCGM-CD medium were normal (S2 Figure).

### Comparison of Gene Expression Profiles

**Figure 5:**
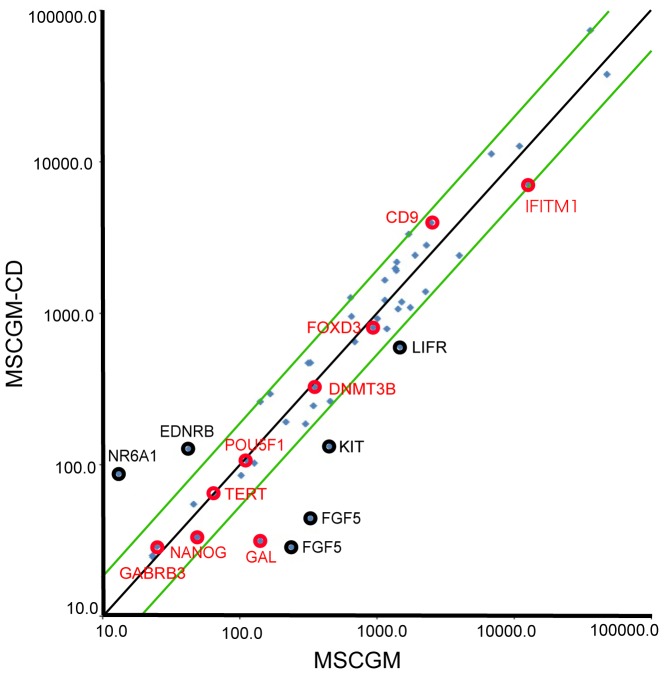
Microarray analysis of gene expression. Scatter plots compared the embryonic stem cell marker genes expression between chemically defined culture conditions (MSCGM-CD) and normal culture conditions (MSCGM) determined by DNA microarray. The green lines indicate the diagonal and 2-fold changes between the two samples. Black and red circles indicate the expression levels of some embryonic stem cell marker genes (S3 Table).

To identify genes that were modulated by the culture conditions, we performed cDNA microarray analysis on 3 DPC pairs (DP245, DP264, and DP265) that had been cultured in MSCGM or MSCGM-CD medium. Genes analyzed, 84 were downregulated more than two-fold in MSCGM-CD compared with MSCGM. In contrast, 51 genes were upregulated more than two-fold in MSCGM-CD compared with MSCGM. However, embryonic stem cell markers related to iPS generation, such as POU5F1 and NANOG, were not differentially expressed between MSCGM and MSCGM-CD conditions (Fig. 5 and S3 Table). Genes significantly upregulated or downregulated more than three-fold in all 3 samples are listed in S4 Table.

## Discussion

Stem cells are invaluable tools for screening, studying mechanisms of diseases, and can potentially serve as a resource for regenerative therapies. Given the wide clinical potential that DPCs possess, our objective was to design a new protocol for the isolation and maintenance of clinical grade cells using chemically defined reagents. This is since other traditional DPC culture protocols use xenogenic products such as FBS, which may impair or damage cell growth, or potentially damage cell lines due to endotoxins and mycoplasma contamination. Furthermore, use of 20% FBS in human mesenchymal stromal cell culture is known to elicit an immune reaction in patients [21] [22].

In this study, we successfully isolated hDPCs using the chemically defined (MSCGM-CD) medium, although their colony forming ability was lower than that observed with ‘conventional’ FBS containing (MSCGM) medium. In early passage cultures (less than passage 3), cells from individual donors grew exponentially in MSCGM-CD medium, although there was variation in growth rates of cells isolated from different donors; two out of three donor-derived cell lines grew slower than in MSCGM. Reduction in cell growth rate became apparent in MSCGM-CD medium in later passages. The cell line-dependent growth rate suggests that each cell line has its own optimum requirements for levels of nutrients including growth factors and/or adhesion molecules. However, cells isolated during culture in MSCGM-CD differentiated toward the odontoblastic cell lineage *in vitro* and formed dentin-like structures when transplanted into immunodeficient mice; this identical to the behavior of cells isolated in MSCGM. Such *in vitro* and *in vivo* differentiation capacity suggests that although MSCGM-CD condition has less potential in the cell expansion stage, this medium allows the maintenance of stem cell like characteristics.

We previously reported that retroviral transduction of four transcription factors (*OCT3/4*, *SOX2*, *KLF4*, and *c-MYC*) can reprogram hDPCs into iPSCs [14]. Other groups also established iPSCs from different types of dental resources such as apical papilla [23], pulp of exfoliated deciduous teeth [23] [24], and wisdom teeth [25]. Human iPSCs are a potential source of patient-specific pluripotent stem cells that could be used to treat a number of human degenerative diseases without evoking immune rejection. Many major challenges, including immunogenicity and the use of oncogenes and retroviruses in the reprogramming of iPSCs need to addressed before hESCs and iPSCs can be safely used as a source for clinical cell therapy [26]. Chief among these is the exposure to undefined animal-derived products during *in vitro* establishment and expansion of the cells. Beltrão-Braga et al. reported iPSC induction under feeder-free conditions on matrigel-coated dishes [24]. We examined the effects of MSCGM-CD medium on the generation of human iPSCs from DPCs using a Sendai virus system [16] [17], and found that the reprogramming efficiency was similar to that obtained using MSCGM medium.

Our microarray analyses showed that the expression of ESC markers was similar, but not identical, between cells grown in MSCGM and MSCGM-CD. Among the embryonic stem cell marker genes defined in *ISCI* [27], *NR6A1*, and *EDNRB* were upregulated in MSCGM-CD medium. In contrast, expression of *GAL*, *LIFR*, *KIT*, and *FGF5* mRNA were higher in MSCGM medium. However, the expression of most embryonic stem cell markers (including *GABRB3*, *TERT*, *POU5F1*, *DNMT3B*, *FOXD3*, *CD9*, *GAL*, and *IFITM1*) was similar in cells grown in both MSCGM-CD and MSCGM media, (Fig. 5, red circles). Expression of these genes is tightly correlated with that of NANOG, a key transcription factor that maintains pluripotency, while *NR6A1*, *EDNRB*, *FGF5*, *KIT*, and *LIFR* (Fig. 5 black circles) have weaker correlations [27]. This may in part explain why growth in MSCGM-CD medium did not affect the iPS induction rate.

In conclusion, our data indicate that MSCGM-CD medium is a valid substitute for MSCGM, as it favors odontoblastic cell differentiation and iPS cell generation. However, MSCGM-CD is not optimal for primary cell growth or long-term propagation of the cells.

## Supporting Information

S1 Figure **Morphology and growth capability of hDPCs cultured in MSCGM or STK2 media.** (A) Images of hDPCs (DP224) cultured for 7 days after seeding, and stained with crystal violet. Cells were obtained from the same donor and cultured with MSCGM or STK2, respectively (n = 5 donors). Scale bar = 400 µm. (B) Number of colonies derived from cells cultured in MSCGM or STK2 medium. hDPCs grown in MSCGM showed significantly higher primary colony formation than those grown in STK2 medium.
(TIF)

S2 Figure **G-banding chromosome analysis.** (A-D) Normal karyotype (46, XX) was observed in DP264 cultured in MSCGM or MSCGM-CD medium and iPSCs generated from cells cultured in MSCGM-CD or MSCGM.
(TIF)

S3 Figure **Expression of osteoblastic differentiation markers in hDPCs cultured in MSCGM or MSCGM-CD media.** hDPCs were cultured in MSCGM or MSCGM-CD and expression levels of *BGLAP*, *RUNX2*, and *SPP1* mRNA were assessed by real-time PCR analyses. mRNA values were divided by those of *GAPDH* and used to calculate expression coefficients. Error bars indicate the SD calculated from triplicates. *P<0.05 compared with MSCGM.
(TIF)

S1 Table **RT-PCR primer pairs.**
(DOCX)

S2 Table **Summary of iPS Clones Established During this Study.**
(DOCX)

S3 Table **Embryonic Stem Cell Marker Genes Upregulated in MSCGM-CD medium.**
(DOCX)

S4 Table **Genes Upregulated (MSCGM-CD>MSCGM) or Downregulated (MSCGM-CD<MSCGM) by 3-fold.**
(DOCX)

S1 Text **Supporting Method and Supporting References.**
(DOCX)
