# Correction: Derivation of iPSCs after Culture of Human Dental Pulp Cells under Defined Conditions

**DOI:** 10.1371/journal.pone.0121771

**Published:** 2015-03-25

**Authors:** The PLOS ONE Staff

The accession number that gives access to the Microarray data in the last sentence of the cDNA Microarray Analysis section of the Materials and Methods is incorrect. The correct accession number is: GSE59493.
