# Supplementary figures and images for: Derivation of iPSCs after Culture of Human Dental Pulp Cells under Defined Conditions
Source: PLoS One. 2014 Dec 18;9(12):e115392. doi: 10.1371/journal.pone.0115392 (PMC4270765; doi:10.1371/journal.pone.0115392)

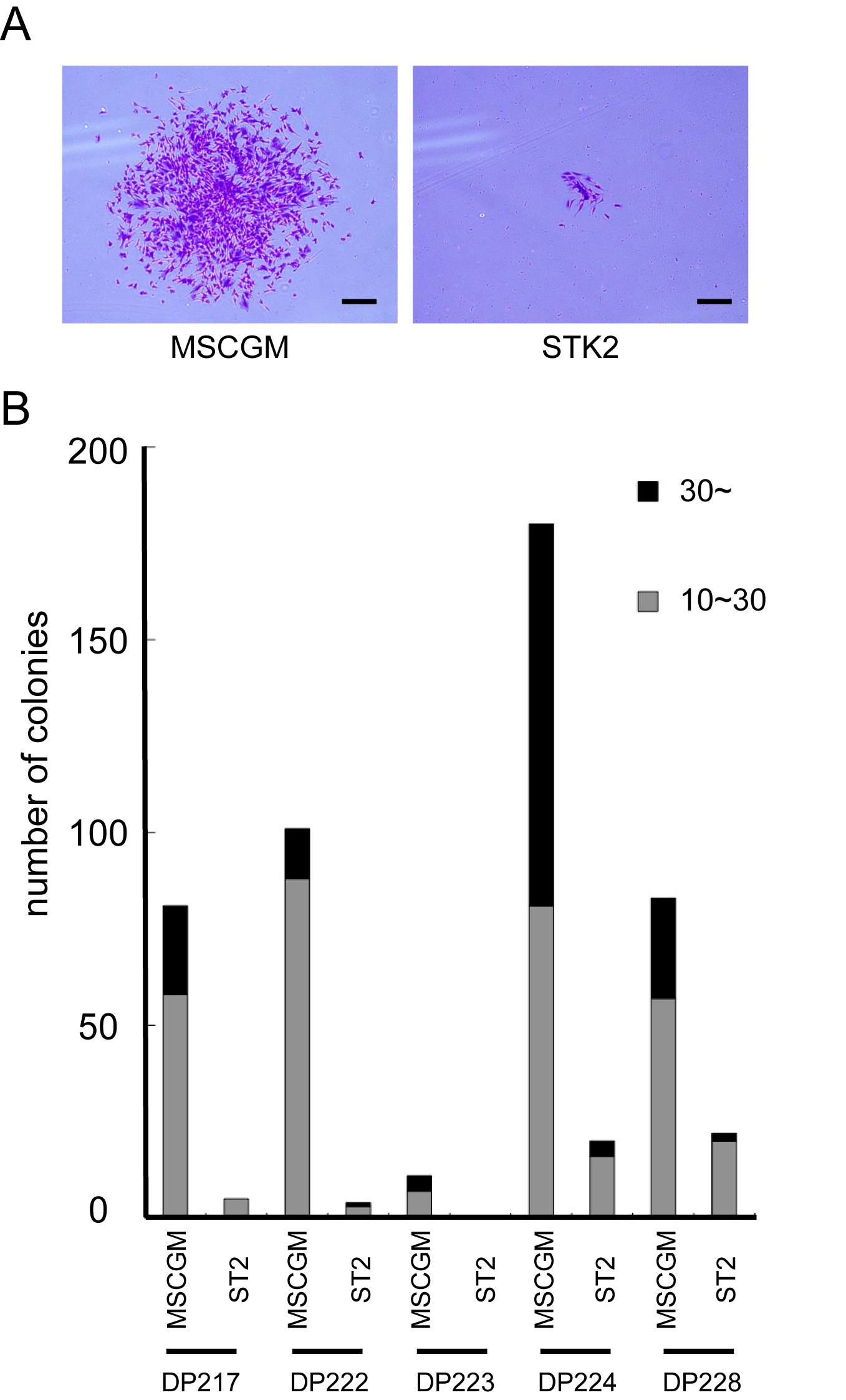

Supplement: S1 Figure — Morphology and growth capability of hDPCs cultured in MSCGM or STK2 media. (A) Images of hDPCs (DP224) cultured for 7 days after seeding, and stained with crystal violet. Cells were obtained from the same donor and cultured with MSCGM or STK2, respectively (n = 5 donors). Scale bar = 400 µm. (B) Number of colonies derived from cells cultured in MSCGM or STK2 medium. hDPCs grown in MSCGM showed significantly higher primary colony formation than those grown in STK2 medium. (TIF) [file pone.0115392.s001.tif]

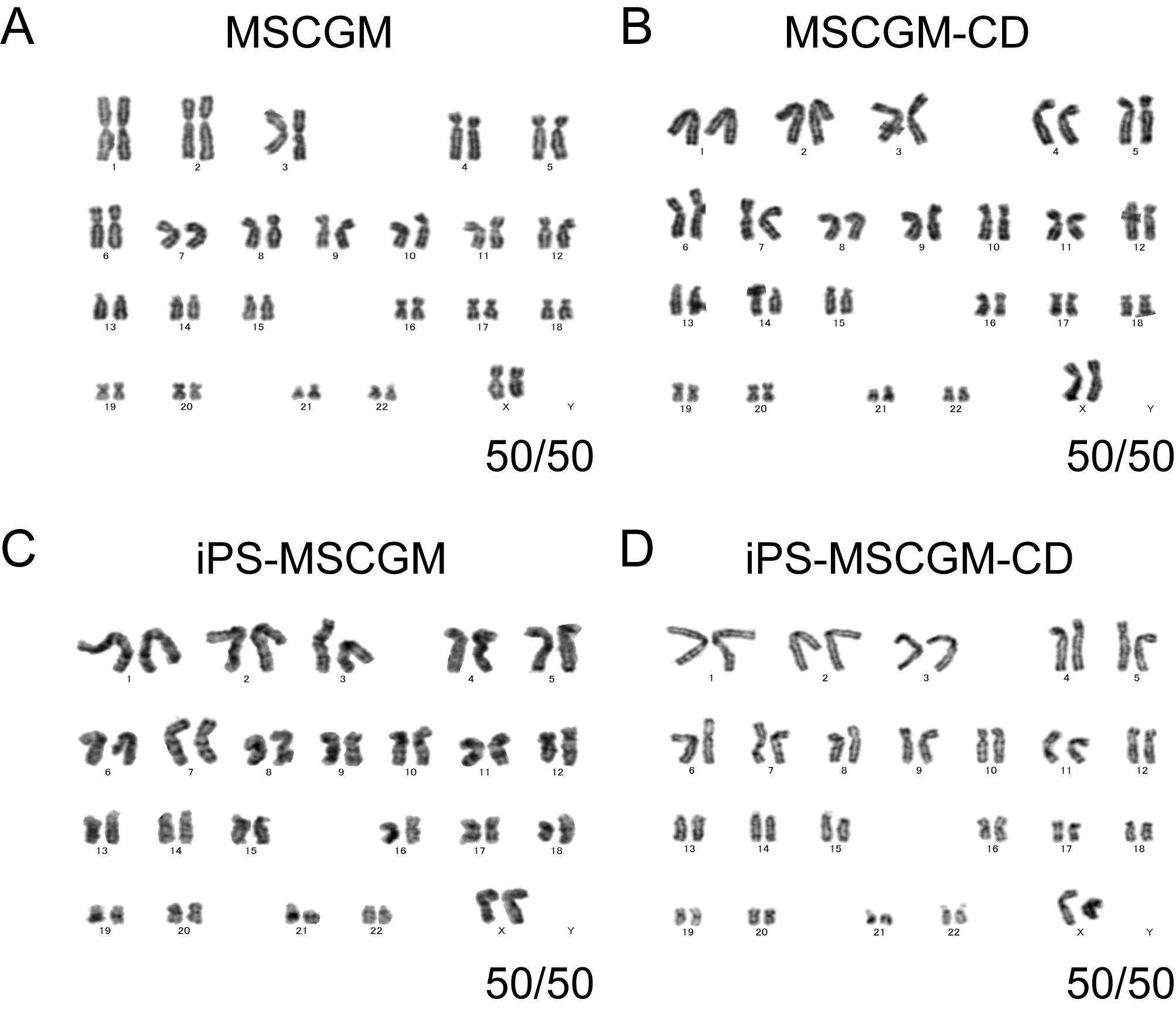

Supplement: S2 Figure — G-banding chromosome analysis. (A-D) Normal karyotype (46, XX) was observed in DP264 cultured in MSCGM or MSCGM-CD medium and iPSCs generated from cells cultured in MSCGM-CD or MSCGM. (TIF) [file pone.0115392.s002.tif]

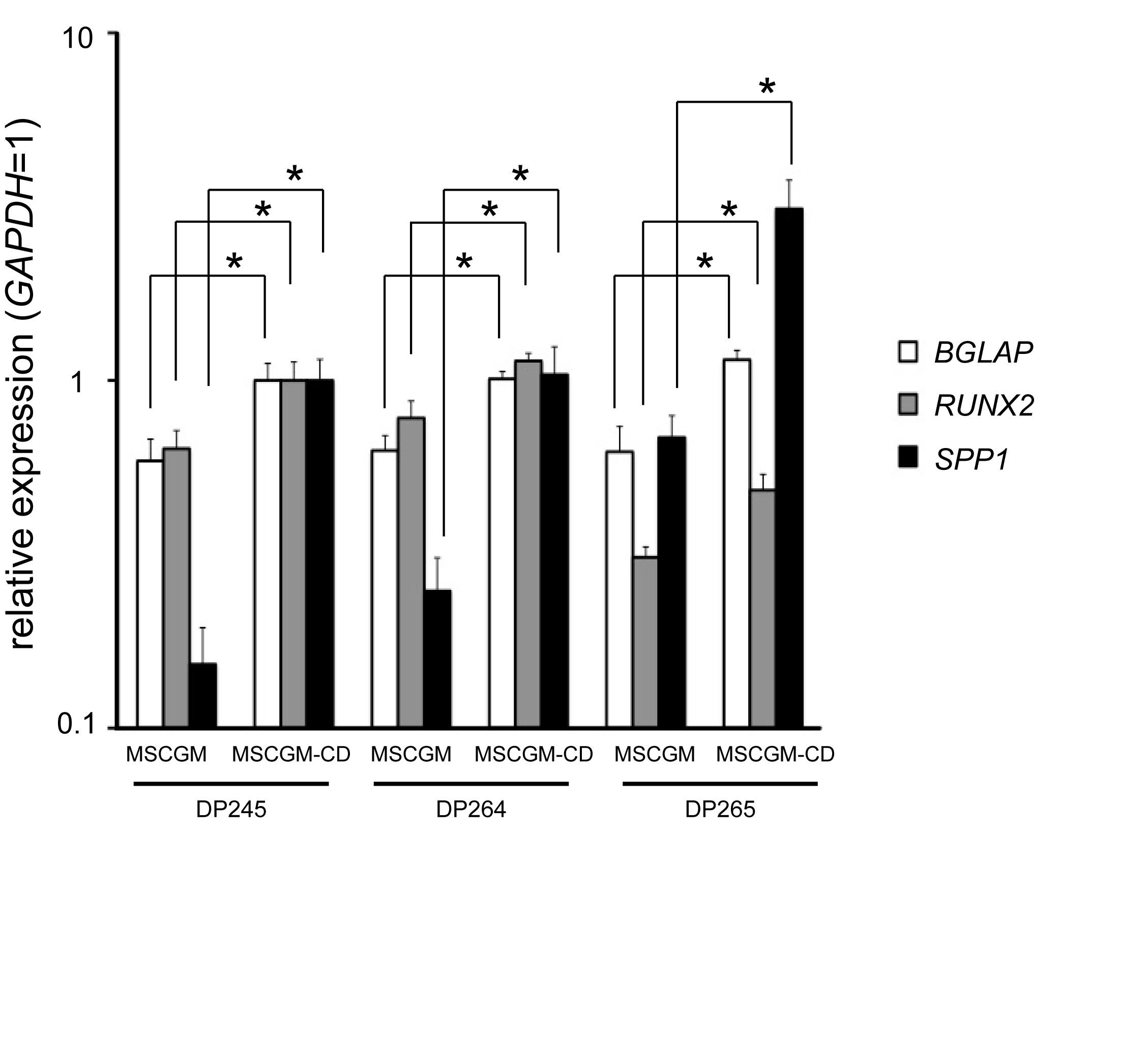

Supplement: S3 Figure — Expression of osteoblastic differentiation markers in hDPCs cultured in MSCGM or MSCGM-CD media. hDPCs were cultured in MSCGM or MSCGM-CD and expression levels of BGLAP, RUNX2, and SPP1 mRNA were assessed by real-time PCR analyses. mRNA values were divided by those of GAPDH and used to calculate expression coefficients. Error bars indicate the SD calculated from triplicates. *P<0.05 compared with MSCGM. (TIF) [file pone.0115392.s003.tif]
