# Supplementary material for: Derivation of iPSCs after Culture of Human Dental Pulp Cells under Defined Conditions
Source: PLoS One. 2014 Dec 18;9(12):e115392. doi: 10.1371/journal.pone.0115392 (PMC4270765; doi:10.1371/journal.pone.0115392)
Supplement: S1 Table — RT-PCR primer pairs. (DOCX) [file pone.0115392.s004.docx]

**Table S1**. RT-PCR primer pairs.

| Target | Sequence (5' to 3') | GenBank Accession Number |
| --- | --- | --- |
| *NANOG*(S)  (AS) | TCCAACATCCTGAACCTCAGCTA  AGTCGGGTTCACCAGGCATC | NM_024865.2 |
| *OCT3/4*(S)  (AS) | GACAGGGGGAGGGGAGGAGCTAGG  CTTCCCTCCAACCAGTTGCCCCAAAC | NM_002701 |
| *SOX2*(S)  (AS) | GGGAAATGGGAGGGGTGCAAAAGAGG  TTGCGTGAGTGTGGATGGGATTGGTG | NM_003106 |
| *REX1*(S)  (AS) | GTCAAATAACCTGAAAGCCCACATC  AATCAATGAGGCATGTTTGTCACTG | NM_174900.3 |
| *KLF4*(S)  (AS) | ACGATCGTGGCCCCGGAAAAGGACC  TGATTGTAGTGCTTTCTGGCTGGGCTCC | NM_004235 |
| *BGLAP*(S)  (AS) | CCCAGGCGCTACCTGTATCAA  GGTCAGCCAACTCGTCACAGTC | NM_199173 |
| *RUNK2*(S)  (AS) | ATGTGTGTTTGTTTCAGCAGCA  TCCCTAAAGTCACTCGGTATGTGTA | NM_001024630 |
| *SPP1*(S)  (AS) | ACACATATGATGGCCCGAGGTGA  TGTGAGGTGATGTCCTCGTCTGTAG | NM_001040058.1 |
| *GAPDH*(S)  (AS) | GCACCGTCAAGGCTGAGAAC  TGGTGAAGACGCCAGTGGA | NM_002046.3 |

Abbreviations: *OCT3/4*, *SOX2*, and *KLF4* primers specifically for endogenous genes; S, sense primers; AS, antisense primers.
