# Supplementary material for: Derivation of iPSCs after Culture of Human Dental Pulp Cells under Defined Conditions
Source: PLoS One. 2014 Dec 18;9(12):e115392. doi: 10.1371/journal.pone.0115392 (PMC4270765; doi:10.1371/journal.pone.0115392)
Supplement: S2 Table — Summary of iPS Clones Established During this Study. (DOCX) [file pone.0115392.s005.docx]

**Table S2.** Summary of iPS Clones Established During this Study.

| Clone | ALP staining | Immunostaining | Real-time PCR | Teratoma |
| --- | --- | --- | --- | --- |
| DP264-CD-iPS  DP264-M-iPS  DP265-CD-iPS  DP265-M-iPS | ✓  ✓ | ✓  ✓  ✓  ✓ | ✓  ✓  ✓  ✓ | ✓  ✓  ✓  ✓ |

Various *in vitro* and *in vivo* assays performed with each iPS clone are indicated by “✓.” Immunostaining is for markers for undifferentiated human ESCs (SSEA-4, TRA1-60, and TRA1-81) and a marker for differentiated ESCs (SSEA-1). Real-time PCR was used to detect ESC-markers *NANOG*, Endo-*OCT3/4*, Endo-*SOX2*, *REX1*, and Endo-*KLF4*. M-iPS, iPS derived from cells cultured in MSCGM medium; CD-iPS, iPS derived from cells cultured in MSCGM-CD medium.
