# Supplementary material for: Derivation of iPSCs after Culture of Human Dental Pulp Cells under Defined Conditions
Source: PLoS One. 2014 Dec 18;9(12):e115392. doi: 10.1371/journal.pone.0115392 (PMC4270765; doi:10.1371/journal.pone.0115392)
Supplement: S3 Table — Embryonic Stem Cell Marker Genes Upregulated in MSCGM-CD medium. (DOCX) [file pone.0115392.s006.docx]

**Table S3.** Embryonic Stem Cell Marker Genes Upregulated in MSCGM-CD medium

| Gene Symbol | Probe Name | Fold Increase |
| --- | --- | --- |
| BRIX1 | A_23_P252855 | 0.78 |
| BRIX1 | A_23_P252857 | 0.75 |
| CCL2 | A_23_P89431 | 0.58 |
| CD9 | A_23_P76364 | 1.60 |
| COMMD3 | A_23_P138514 | 1.16 |
| CRABP2 | A_23_P115064 | 1.45 |
| CTNNB1 | A_23_P29495 | 1.66 |
| CTNNB1 | A_24_P608330 | 1.85 |
| CTNNB1 | A_23_P29499 | 1.98 |
| DCN | A_23_P64873 | 2.06 |
| DIAPH2 | A_32_P50834 | 1.96 |
| DIAPH2 | A_23_P85004 | 1.76 |
| DNMT3B | A_23_P28953 | 0.92 |
| EDNRB | A_23_P2831 | 0.94 |
| EDNRB | A_24_P330263 | 3.00 |
| FGF5 | A_24_P401855 | 0.12 |
| FGF5 | A_23_P212800 | 0.13 |
| FOXD3 | A_23_P46560 | 0.86 |
| GABRB3 | A_23_P14821 | 1.16 |
| GAL | A_23_P374844 | 0.22 |
| GRB7 | A_23_P163992 | 0.83 |
| IFITM1 | A_23_P72737 | 0.56 |
| IFITM2 | A_24_P287043 | 0.80 |
| IGF2BP2 | A_23_P250156 | 1.07 |
| IGF2BP2 | A_23_P95672 | 0.80 |
| IL6ST | A_32_P223777 | 0.62 |
| IL6ST | A_32_P140656 | 0.61 |
| IL6ST | A_23_P502470 | 0.62 |
| IL6ST | A_32_P45168 | 0.60 |
| KIT | A_23_P110253 | 0.29 |
| LIFR | A_24_P325992 | 0.41 |
| LIFR | A_24_P397386 | 0.69 |
| NANOG | A_23_P204640 | 0.68 |
| NGFR | A_23_P389897 | 1.19 |
| NR5A2 | A_23_P161004 | 1.05 |
| NR5A2 | A_24_P920090 | 1.09 |
| NR6A1 | A_24_P860842 | 6.66 |
| NUMB | A_23_P88381 | 0.91 |
| POU5F1 | A_23_P59138 | 0.95 |
| PTEN | A_23_P98085 | 1.38 |
| PTEN | A_24_P252785 | 1.23 |
| PTEN | A_24_P913115 | 1.56 |
| REST | A_23_P167081 | 1.44 |
| REST | A_24_P246787 | 1.27 |
| SEMA3A | A_24_P192301 | 0.71 |
| SEMA3A | A_23_P317591 | 0.67 |
| SFRP2 | A_23_P81103 | 1.49 |
| SFRP2 | A_24_P934546 | 1.46 |
| SFRP2 | A_24_P137501 | 1.45 |
| TERT | A_23_P110851 | 0.96 |
| TH | A_24_P924602 | 0.88 |
