# Supplementary material for: Derivation of iPSCs after Culture of Human Dental Pulp Cells under Defined Conditions
Source: PLoS One. 2014 Dec 18;9(12):e115392. doi: 10.1371/journal.pone.0115392 (PMC4270765; doi:10.1371/journal.pone.0115392)
Supplement: S4 Table — Genes Upregulated (MSCGM-CD>MSCGM) or Downregulated (MSCGM-CD<MSCGM) by 3-fold. (DOCX) [file pone.0115392.s007.docx]

**Table S4.** Genes Upregulated (MSCGM-CD>MSCGM) or Downregulated (MSCGM-CD<MSCGM) by 3-fold

| Gene Symbol | MSCGM-CD>MSCGM  GenBank Accession Number | Fold Increase | Gene Symbol | MSCGM-CD<MSCGM  GenBank Accession Number | Fold Increase |
| --- | --- | --- | --- | --- | --- |
| BEAN1 | A_32_P153195 | 118.31 | IGFBP5 | A_23_P383009 | 92.52 |
| CACNA1G | A_23_P107247 | 35.02 | FST | A_23_P110531 | 27.61 |
| VAV3 | A_23_P201551 | 17.48 | KLHDC7B | A_24_P117410 | 11.69 |
| PCDH9 | A_23_P420236 | 9.98 | HSPA5 | A_24_P18190 | 10.56 |
| OGDHL | A_23_P161297 | 9.68 | IL12A | A_23_P91943 | 8.94 |
| MLPH | A_23_P165783 | 8.20 | CHDH | A_23_P69293 | 6.33 |
| JAKMIP2 | A_23_P156390 | 4.54 | MANF | A_23_P132793 | 5.52 |
| COL6A1 | A_24_P331918 | 4.52 | UGCG | A_23_P313389 | 5.12 |
| GLI3 | A_23_P111531 | 4.23 | HERPUD1 | A_23_P54846 | 4.83 |
| CKMT2 | A_23_P144778 | 3.74 | FICD | A_23_P128246 | 4.6 |
| ZNF365 | A_24_P226970 | 3.47 | CRELD2 | A_23_P33465 | 4.47 |
| MC1R | A_23_P329271 | 3.44 | SLC37A1 | A_23_P17695 | 4.26 |
| HTRA1 | A_23_P97990 | 3.35 | PDIA4 | A_23_P42802 | 4.21 |
| HMGCS1 | A_23_P133263 | 3.27 | CEACAM19 | A_23_P78518 | 4.15 |
| NTNG2 | A_23_P423331 | 3.13 | PVR | A_24_P65616 | 3.95 |
| RERG | A_23_P204296 | 3.04 | TMEM50B | A_23_P57304 | 3.81 |
| ACAT2 | A_23_P383835 | 3.02 | DYSF | A_23_P39931 | 3.37 |
| KIAA0408 | A_23_P215048 | 3.01 | HSP90B1 | A_23_P2601 | 3.35 |
|  |  |  | NUCB2 | A_24_P595460 | 3.30 |
|  |  |  | PLCB4 | A_23_P28898 | 3.20 |
|  |  |  | LMAN1 | A_23_P78342 | 3.12 |
