# Supplementary material for: Derivation of iPSCs after Culture of Human Dental Pulp Cells under Defined Conditions
Source: PLoS One. 2014 Dec 18;9(12):e115392. doi: 10.1371/journal.pone.0115392 (PMC4270765; doi:10.1371/journal.pone.0115392)
Supplement: S1 Text — Supporting Method and Supporting References. (DOCX) [file pone.0115392.s008.docx]

**Supporting Information Method**

**Cytogenetic analysis**

For cytogenetic analysis, preparations of metaphase chromosomes from hDPCs at passage 7 and iPSCs were obtained and were banded with Giemsa-trypsin (Nishio *et al*., 2001). A detailed karyotype was analyzed in 50 cells of each hDPC and iPSC line. Karyotypes were described using the short version of the International System for Human Cytogenetic Nomenclature (Shaffer *et al*., 2013).

**Supporting Information References**

Nishio J, Iwasaki H, Ishiguro M, Ohjimi Y, Yo S, et al. (2001) Supernumerary ring chromosome in a Bednar tumor (pigmented dermatofibrosarcoma protuberans) is composed of interspersed sequences from chromosomes 17 and 22: a fluorescence in situ hybridization and comparative genomic hybridization analysis. Genes Chromosomes Cancer 30: 305–309.

Shaffer LG, editors (2013) ISCN. An international system for human cytogenetic nomenclature. Basel: S Karger.
